# The Effect of Changes in the Aging Temperature Combined with Deep Cryogenic Treatment on the Structure, Phase Composition, and Micromechanical Properties of the WE43 Magnesium Alloy

**DOI:** 10.3390/ma16237447

**Published:** 2023-11-30

**Authors:** Adrian Barylski, Krzysztof Aniołek, Grzegorz Dercz, Izabela Matuła, Jan Rak, Izabela Mazur

**Affiliations:** Faculty of Science and Technology, Institute of Materials Engineering, University of Silesia in Katowice, 75 Pułku Piechoty Street 1A, 41-500 Chorzów, Poland; krzysztof.aniolek@us.edu.pl (K.A.); grzegorz.dercz@us.edu.pl (G.D.); izabela.matula@us.edu.pl (I.M.); jan.rak@us.edu.pl (J.R.); iza.mazur.16@gmail.com (I.M.)

**Keywords:** WE43 magnesium alloy, solution treatment, aging, deep cryogenic treatment, microstructure, XRD, EDS, instrumental indentation

## Abstract

This paper examines the optimal aging temperature of WE43 alloy that has undergone precipitation hardening in conjunction with deep cryogenic treatment. The microstructure and phase composition were investigated, a microanalysis of the chemical composition was performed, and instrumental indentation tests were performed to determine the parameters of the micro-mechanical properties of the alloy after different heat treatment variants. It has been proven that a decrease in the aging temperature from 250 °C to 225 °C and the introduction of a deep cryogenic treatment lead to favorable changes in the microstructure of the alloy (reduction in grain size, increase in the number, and change in the type of β-phase precipitates). The changes in the alloy structure achieved by lowering the aging temperature contribute to the improvement of the micromechanical properties of the test material. The most advantageous results were recorded for an alloy subjected to solution treatment and aged at 225 °C for 24 h with deep cryogenic treatment: a 30% increase in hardness, a 10% increase in Young’s modulus, an improvement in elastic properties, and increased resistance to deformation of the alloy were shown compared to the initial (as-received) state. Raising the aging temperature to 250 °C leads to a phenomenon known as alloy overaging for both alloys after classical precipitation hardening and after deep cryogenic treatment. The results indicate the significant effectiveness of the proposed heat treatment in improving the service life of the Mg-Y-Nd-Zr (WE43) alloy.

## 1. Introduction

Magnesium alloys, as lightweight (four times lighter than steel), rigid, and high-strength materials, have low resistance to high temperatures. To enable their use in the automotive, space, and aviation industries, it was necessary to introduce alloying additions, such as rare-earth metals [1]. The WE43 alloy contains zirconium, neodymium, and yttrium. Zirconium improves the corrosion and plastic properties of alloys and inhibits grain growth during heat treatment. Neodymium and yttrium, on the other hand, contribute to the formation of high-strength intermetallic phases that strengthen alloys both dispersively and solutionally [2].

The WE43 alloy can be obtained using various methods, such as pressure casting, continuous casting, or extrusion. Depending on the method used, its initial structure and properties in the initial state may vary. Its low density (approx. 1.8 g/cm^3^), yield stress (190–250 MPa), tensile strength (250–300 MPa), which is also retained at elevated temperatures, and higher resistance to galvanic corrosion as compared to other magnesium alloys facilitate using the WE43 in technical solutions wherever gearbox castings, racing car wheels, or components of engines or chassis are manufactured [1].

Magnesium alloys with rare-earth metals, including the WE43, are also used in biomedicine, especially in orthopaedics, where components such as nails, self-tapping screw tips, cannulated screws, and interference screws used to fix grafts in the tibia or femur are manufactured from them. Magnesium—rare earth alloys can also be used in the treatment of the cardiovascular system; e.g., in stents [3,4]. An additional advantage is their high biocompatibility (mechanical properties similar to those of the cortical bone prevent stress shielding effect), bioactivity (when decomposing in the body, the alloy creates an alkaline environment which inhibits, inter alia, the growth of bacteria supporting the wound healing process, and has excellent osteoconductive properties), biodegradability (implants have a stability comparable to that of conventional materials, such as steel or titanium, but are reabsorbable, i.e., they stabilize the bones until they can carry sufficient load on their own and then the implants are gradually absorbed by the body, which prevents, among other things, costly and painful implant removal procedures) [5,6,7,8,9,10].

The main disadvantages of magnesium-rare earth alloys are: low yield stress, especially at elevated temperatures; poor corrosion resistance in alkaline environments; and poor resistance to tribological wear. The WE43 magnesium alloy is the object of a number of scientific research studies due to its high potential for applications in high-tech industries. Research on magnesium alloys mainly focuses on the purification of the alloys (Cu in amounts exceeding 0.05% wt. and Fe and Ni in amounts exceeding 0.005% wt. are the most harmful impurities affecting the corrosion resistance and plasticity of the alloy) [11], optimization of its chemical composition [12,13,14], preparation methods [2], heat treatment [15,16,17,18,19,20], plastic forming [21,22], and surface modification, including chromium plating, TiN, CrN, AlN aluminum, and titanium coatings; and coatings made of calcium-phosphate compounds (Ca-P) to obtain better mechanical, corrosion, and tribological properties [23].

One of the methods for modifying the properties of the alloy is the precipitation hardening process [15,16], which, with appropriately selected time and temperature, develops the desirable properties of solid magnesium alloys by changing the mechanical properties in a wide range [10,16]. Deep cryogenic treatment (DCT), on the other hand, involves cooling and sub-zero treatment of the alloy at liquid nitrogen temperature (−196 °C), followed by reheating. DCT improves both mechanical and tribological properties while at the same time reducing the stresses present in the alloy. It is used for steels, aluminum alloys, and magnesium alloys [24,25,26,27,28,29,30]. Cryogenic treatment-induced changes in the microstructure, such as an increase in the number of twins and changes in grain orientation, affect the properties of Al, Gd, and Li magnesium alloys. The concentration of precipitated phases serves to enhance the strength of magnesium alloys. Conversely, the exclusion of brittle phases at low temperatures can improve flexibility, which further advances corrosion resistance. The diffusion transformation phenomenon leads to shrinkage and may lead to the formation of precipitates with less atomic density than the surrounding material, making it an attractive avenue for future research on magnesium alloys. Magnesium alloys exhibit superior tensile strength and ductility, particularly at low temperatures, where twinning, rather than dislocation sliding as with steel, serves as the primary deformation mechanism. At lower temperatures, the solubility of atoms of the alloying elements in the solid state decreases, resulting in the creation of precipitates that enhance the alloy’s strength [31,32,33,34,35].

This paper proposes a combination of deep cryogenic treatment with precipitation hardening, as well as the selection of the aging temperature in this process, so as to obtain the most favorable mechanical properties by modifying the structure. The present study contributes to the state of knowledge on this subject due to the fact that there is a lack of reports on the heat treatment of this complex type in the relevant literature, a rare exception being our previous studies investigating an alloy with a higher yttrium content, WE54 [15,16,17,18]. 

The main objectives of this study were: to select the heat treatment conditions (solution treatment temperature and aging temperature) for the WE43 alloy; to determine the changes in its microstructure induced by precipitation hardening combined with DCT using a metallographic and SEM electron microscope together with a quantitative analysis and EDS microanalysis of the chemical composition; to perform a phase analysis using X-ray diffraction; and to perform micromechanical tests using the microindentation technique.

## 2. Materials and Methods

The material used in experimental tests was the Mg-Y-Nd-Zr alloy (WE43), manufactured by Luxfer MEL Technologies (Manchester, UK). The chemical composition of the alloy in its as-delivered condition is presented in Table 1. 

The alloy was supplied in the form of rods with a diameter φ = 25.4 mm and a length of 1 m. Disc-shaped samples with the nominal diameter of the rod, a diameter of φ = 9 mm, and a sample height *h* = 5 mm were cut for all the tests.

The process of precipitation hardening was conducted in a laboratory muffle furnace (Czylok, Jastrzębie-Zdrój, Poland) in the air atmosphere (the deviation from the rated temperature is ±5 °C). The temperature of the solution heat treatment determined in our previous studies was 545 °C, with a treatment time of 8 h. In order to select the appropriate temperature, the aging process was carried out at four different temperatures: 175 °C, 200 °C, 225 °C, and 250 °C. The time was 24 h. For all variants, a complex heat treatment process was conducted, which consisted of a combination of solution treatment and aging with a deep cryogenic treatment carried out for 24 h at liquid nitrogen temperature (−196 °C). This yielded 12 different variants of test material. Table 2 presents a summary of the treatment carried out, together with a detailed description of the designations for all variants.

Preparation of the test samples was carried out on an automatic polishing machine by grinding with paper of different grit sizes (220, 800, 1200, 2000, and 4000) and polishing with a polishing cloth using a colloidal silica suspension for final polishing (OP-S NonDry by Struers Inc., Cleveland, OH, USA). Samples for microscopic tests were mounted before grinding and polishing. The obtained heat-treated microsections were etched in Nital 3%, while the microsections in the initial state and those after sub-zero treatment were etched in Nital 5% (a nitric acid (V)—HNO_3_/ethyl alcohol solution). The samples were then cleaned in isopropyl alcohol using an ultrasonic cleaner.

Observation of the metallographic specimens and a quantitative description of their microstructures (volume fraction of intermetallic phases—V_V_ [%]; grain size—G; as per ASTM E112 standard [36]; mean grain area—S_A_ [μm^2^]) were performed using an Olympus GX-51 light microscope equipped with a camera and Olympus Stream Essentials 1.7 software (Olympus, Tokyo, Japan). Examination was also performed using a JEOL JSM-6480 scanning electron microscope (Jeol, Tokyo, Japan) equipped with an adapter for X-ray microanalysis with the energy-dispersive X-ray spectroscopy method (EDS, working voltage: 20 kV) (IXRF, Austin, TX, USA). Images were acquired in SE (secondary electrons) mode at a magnification within the range of 30× to 4000×, which allowed an accurate observation of the microstructure and precipitates.

The X-ray diffraction method (XRD) was used to investigate the phase composition of the material with a Philips X`Pert PW 3040/60 diffractometer (Philips PANalytical, Almelo, The Netherlands) using a copper anode tube (λCuKα—1.54178 Ǻ) supplied with a 30 mA current at 40 kV and a diffracted-beam graphite monochromator for a wave length coming from the copper anode. The counting step in the “step scanning” method was 0.04° with a counting time of 25 s/step, in the angular range of 10°–140° 2θ. The divergence slit in the incident and diffracted beam was 1/2°, and 2° Soller slits were also applied. The ICDD Card PDF 4 database was used to analyze the diffractograms obtained.

Instrumental indentation testing (IIT) was performed using the Micro Combi Tester MCT^3^ (Anton Paar, Corcelles-Cormondrèche, Switzerland). A calibrated Berkovich indenter (B-V83) with an angle of α = 65.3° ± 0.3° was used. Indentation curves were registered at a maximum load of F_max_ = 250 mN. Loading and unloading times were determined in accordance with the recommendations of the ISO 14577 standard [37] at 30 s, and the hold time under maximum load was 10 s. For each specimen, 10 indentations were made. Hardness H_IT_ and Young’s modulus E_IT_ were determined using the Oliver-Pharr method [38]. Based on the registered loading-unloading curves, the values of total indentation work, W_tot_, and its components—the work of plastic deformation; W_plast_; and the work of elastic deformation; W_elast_—were determined; along with the percentage of the work of elastic recovery—η_IT_.

## 3. Research Results and Discussion

### 3.1. Examination of the WE43 Alloy Microstructure

**Figure 1:**
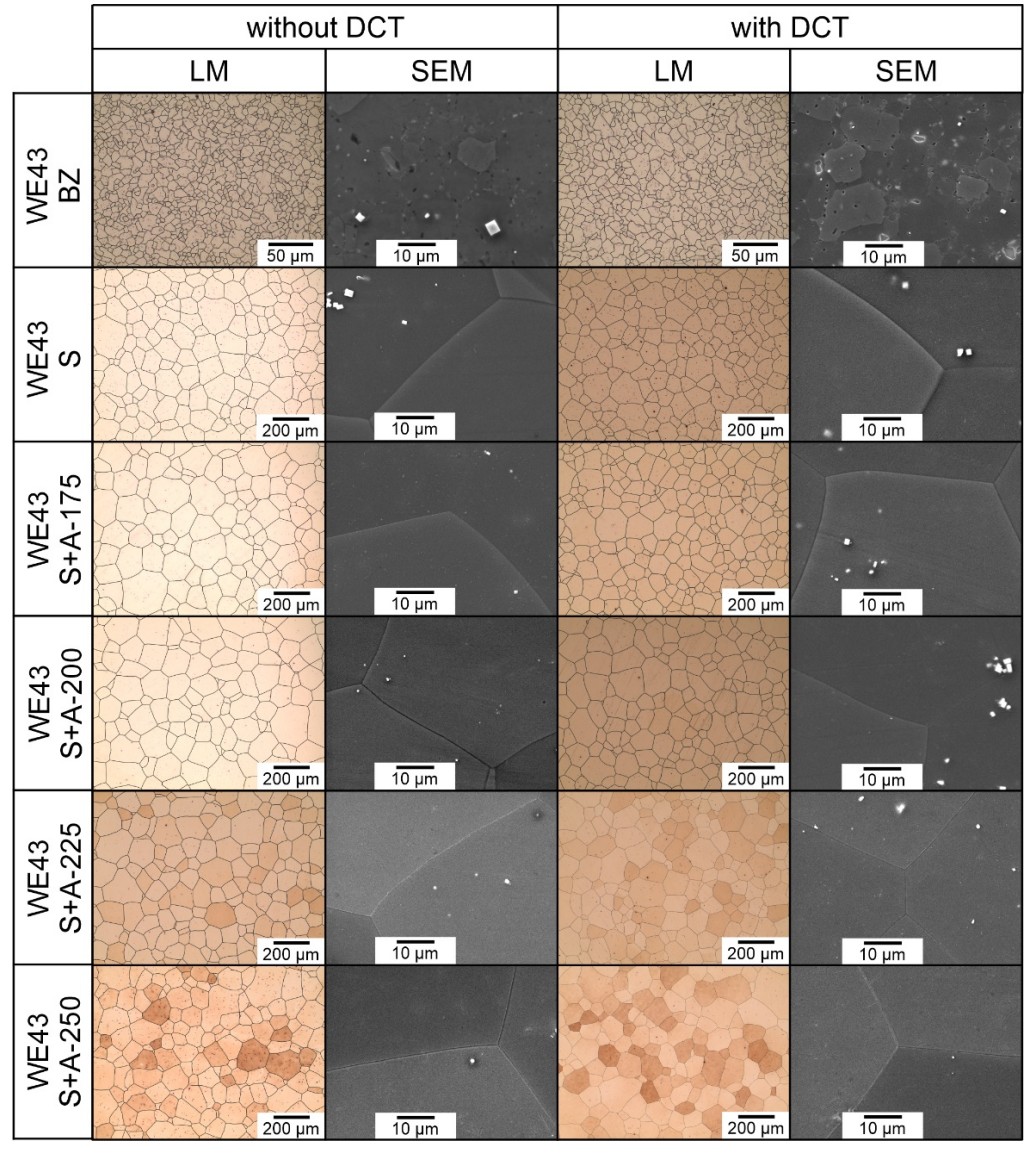
Microstructure of the WE43 magnesium alloy in the initial state (BZ), after deep cryogenic treatment (BZ with DCT), after solution treatment (S), after solution treatment and deep cryogenic treatment (S with DCT), after solution treatment and aging (S + A), and after precipitation hardening combined with deep cryogenic treatment (S + A with DCT) at different aging temperatures: 175 °C, 200 °C, 225 °C, and 250 °C.

**Figure 2:**
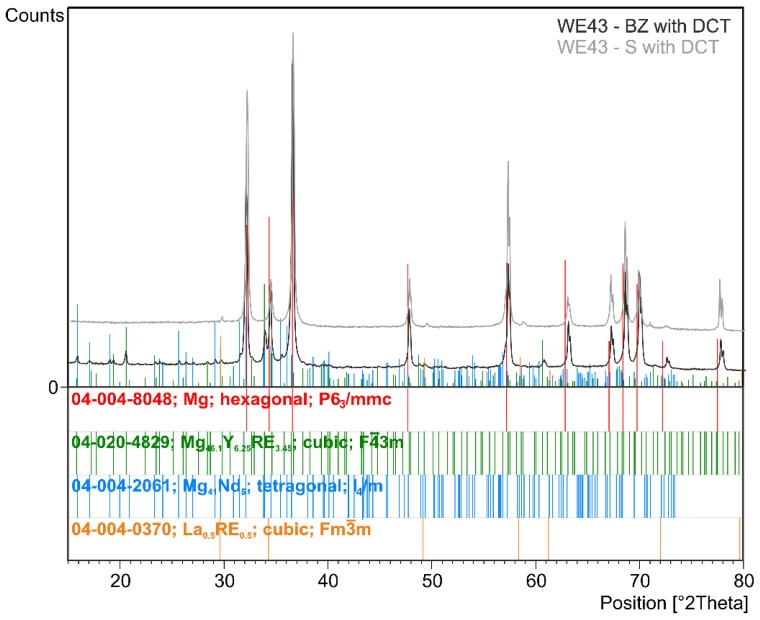
X-ray diffraction pattern (XRD) of the WE43 magnesium alloy after deep cryogenic treatment (BZ with DCT) and after solution treatment and deep cryogenic treatment (S with DCT).

**Figure 3:**
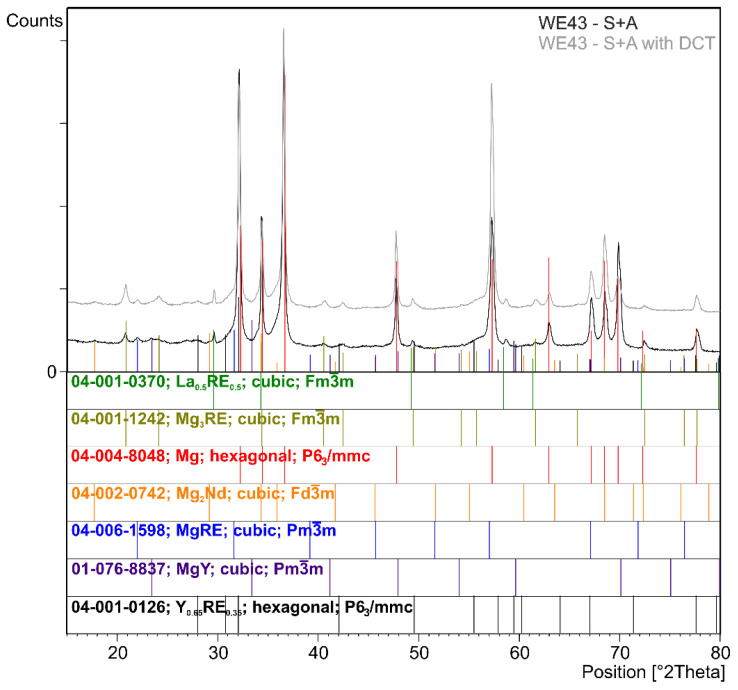
X-ray diffraction pattern (XRD) of the WE43 magnesium alloy after solution treatment and aging (S + A) and after precipitation hardening combined with deep cryogenic treatment (S + A with DCT).

**Figure 4:**
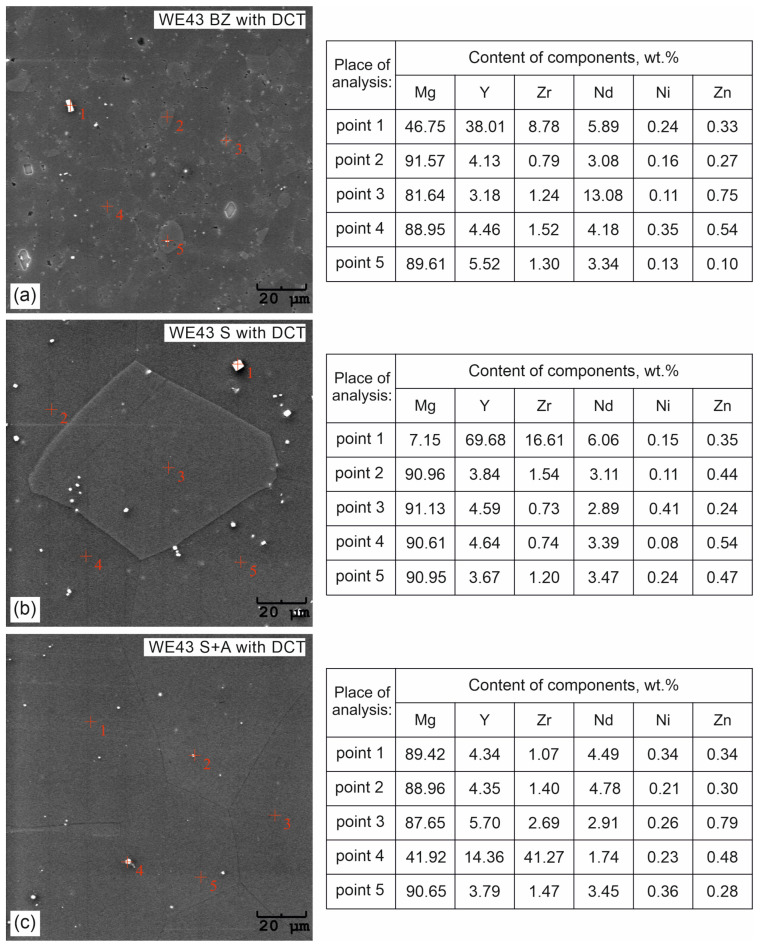
Microanalysis of the chemical composition of the WE43 alloy microstructure after deep cryogenic treatment (BZ with DCT)—(**a**); after solution treatment and deep cryogenic treatment (S with DCT)—(**b**); after precipitation hardening combined with deep cryogenic treatment (S + A with DCT)—(**c**).

**Figure 5:**
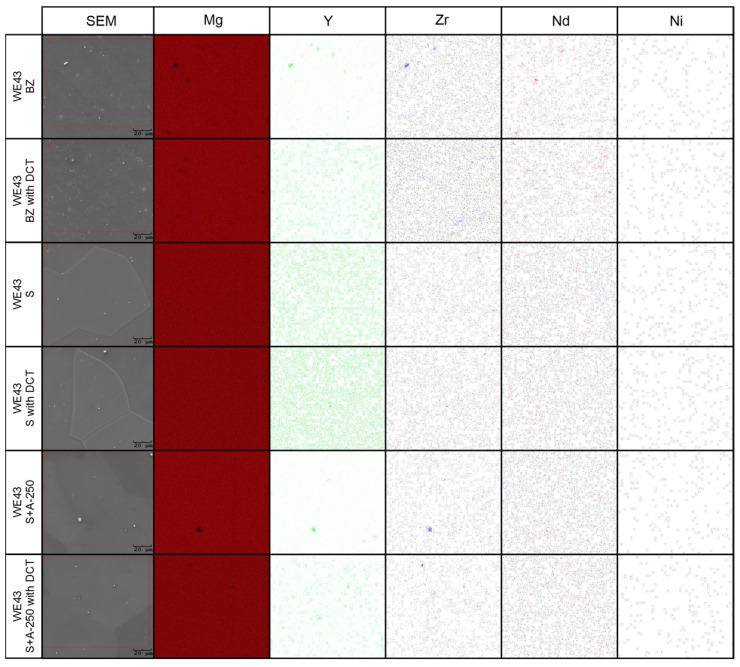
Distributions of map elements of the WE43 alloy in the initial state (BZ), after deep cryogenic treatment (BZ with DCT), after solution treatment (S), after solution treatment and deep cryogenic treatment (S with DCT), after solution treatment and aging (S + A), and after precipitation hardening combined with sub-zero treatment (S + A with DCT).

The effect of changes in the aging temperature on the microstructure of the WE43 alloy in its initial state after precipitation hardening and after a complex heat treatment consisting of solution treatment, cryogenic treatment in liquid nitrogen, and aging is illustrated in Figure 1. The results of quantitative calculations of the microstructure according to the ASTM E112 standard are presented in Table 3. X-ray analysis of the phase composition is presented in Figure 2 and Figure 3. Distributions of map elements in Figure 4 and EDS microanalysis of the chemical composition in Figure 5.

The examined magnesium alloy in its initial state had a fine-grained structure of solid solution α-Mg (ICDD: 04-004-8048) (Figure 1). Analysis of the phase composition (Figure 2) and microscopic observations confirmed the presence of Mg_41_Nd_5_ (ICDD: 04-004-2061), Mg_41_Nd_5_ (ICDD: 04-004-2061)_,_ and Mg_46.1_Y_6.25_RE_3.45_ (ICDD: 04-020-4829) phases. Electron microscope (SEM) images also show precipitates with a characteristic cuboid shape—Mg_24_Y_5_ phases (ICDD: 01-077-9449)—and zirconium-rich areas; the absence of phase Mg_24_Y_5_ reflexes on diffractograms may be due to the fact that its quantity was below the detectability threshold of the method; this may also be influenced by the reduced Y content as compared to the previously studied WE54 alloy [15,16,17,18]. No significant differences were found between the structure of the WE43 alloy in its initial state and after deep cryogenic treatment carried out over a 24-hour period (DCT). A quantitative analysis of the microstructure (Table 3) in the initial state and after sub-zero treatment showed a small mean grain size of 57.8 μm^2^ (G = 11.12; ASTM E112) for the initial sample and 73.5 μm^2^ (G = 10.78; ASTM E112) for the sample after DCT. The volume fraction of intermetallic phases was 9.03% for the initial sample and 7.34% for the sample subjected to sub-zero treatment.

Microanalysis of the phase composition (EDS) (Figure 4) and distribution maps of the elements (Figure 5) confirmed the presence of the following elements in the WE43 alloy: Mg, Y, Zr, Nd, Ni, and Zn. The results were consistent with the manufacturer’s certification, and no additional undesirable elements were identified in the alloy composition.

When analyzing the microstructure of the WE43 alloy after the solution treatment, one can notice, in the first instance, a 120-fold increase in the grain size (Table 3). The alloy subjected to sub-zero treatment after solution treatment, on the other hand, was characterized by an approximately 100-fold increase in the grain area. Quantitative calculations of the microstructure showed that after solution treatment, most of the precipitates dissolved in the matrix and their fraction decreased by 5 times, and in the case of solution treatment combined with sub-zero treatment, by more than 10 times compared to the alloy in the initial state, unlike in the case of the WE54 alloy, which was studied and described in our previous articles, where there was no such an effect [16,17,18]. On XDR diffractograms (Figure 2), α-Mg phases (ICDD: 04-004-8048) and La_0.5_RE_0.5_ (ICDD: 04-001-0370) were identified. Microanalysis of the chemical composition (Figure 4b) and observations of the distributions of map elements (Figure 5) also indicate a homogeneous composition of the WE43 alloy after solution treatment.

**Figure 6:**
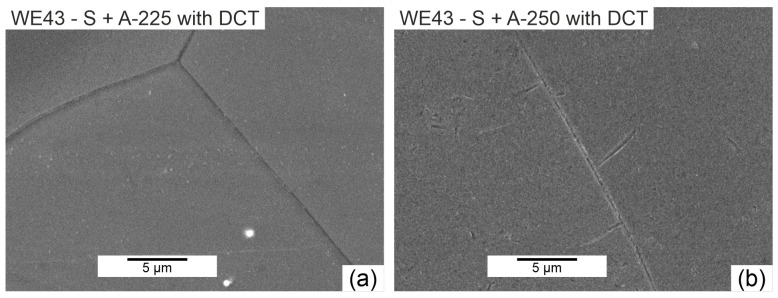
Example of the WE43 alloy precipitates’ microstructure after solution treatment and aging with deep cryogenic treatment: aging temperature 225 °C (S + A-225 with DCT)—(**a**); aging temperature 250 °C (S + A-250 with DCT)—(**b**).

The next step was to investigate the effect of changes in the aging temperature in a complex heat treatment process (solution treatment, aging, and sub-zero treatment). The introduction of cryogenic treatment to the precipitation hardening process for aging temperatures of 175–225 °C/24 h led to a decrease in the grain size compared to samples not subjected to this process. Examining, in turn, samples aged at 250 °C/24 h, it can be observed that the grain size increases significantly in comparison with the 225 °C/24 h parameters for both heat treatment types. When analyzing the effect of the aging temperature on the microstructure, particular attention should be paid to the number and type of β-phase strengthening precipitates. For aging temperatures of 175–225 °C, it was observed that, as the aging temperature increased, there was a maximum 5-fold increase in the number of precipitates compared to the alloy after solution and sub-zero treatment. These precipitates are of fine size, and only such can result in the strengthening of the material. The most advantageous results were obtained for the alloy aged at 225 °C/24 h and subjected to deep cryogenic treatment (S + A-225 with DCT). For the alloy aged at 250 °C/24 h, the highest number of precipitates was observed, but their size and length increased significantly, which, coupled with an increase in the grain size, can indicate the overaging of the alloy (Figure 6). The phases identified during XRD testing were: Mg (ICDD: 04-004-8048), La_0.5_RE_0.5_ (ICDD: 04-001-0370), Mg_3_RE (ICDD: 04-001-1242), Mg_2_Nd (ICDD: 04-002-0742), MgRE (ICDD: 04-006-1598), MgY (ICDD: 01-076-8837), and Y_0.65_RE_0.35_ (ICDD: 04-001-0126). The diffractogram is presented in Figure 3. Microanalysis of the phase composition (EDS) also confirmed the presence of phases including yttrium as well as zirconium-rich areas (Figure 4).

Due to the lower aging temperature of 225 °C, as well as the aging time previously tested and decreased from 48 h to 24 h [16,17], obtained by applying sub-zero treatment, it is possible to support the precipitation process and prevent the re-growth of grains, which adversely affects the properties of magnesium alloys with rare earth metals.

### 3.2. Instrumental Microindentation—Micromechanical Tests

Reducing the aging temperature through advantageous changes in the structure of the WE43 alloy had a significant influence on the changes in micromechanical properties observed in instrumental microindentation tests. Despite the fact that aging offers benefits such as increasing the strength of the alloys, restoring balance in the structure, and eliminating unstable conditions caused by the previous operation (e.g., solution treatment), particular care must be taken to ensure that the aging process is carried out below the solvus equilibrium temperature. If the process is continued beyond a specified time or at an inadequate temperature, the hardness will eventually decrease, which is a phenomenon known as overaging [39].

**Figure 7:**
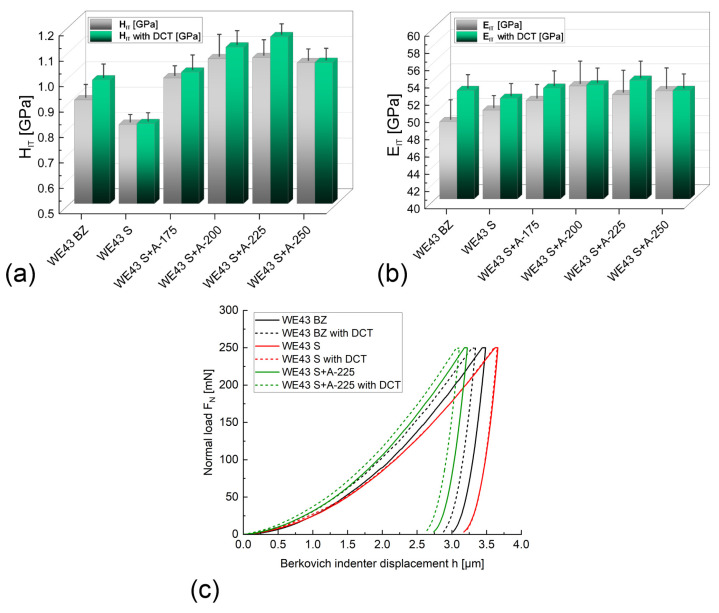
Hardness H_IT_—(**a**); Young’s modulus E_IT_—(**b**); and indentation curves—(**c**) of the as-received WE43 alloy after deep cryogenic treatment and after solution treatment with aging at temperatures of 175–250 °C (with and without cryogenic treatment).

**Figure 8:**
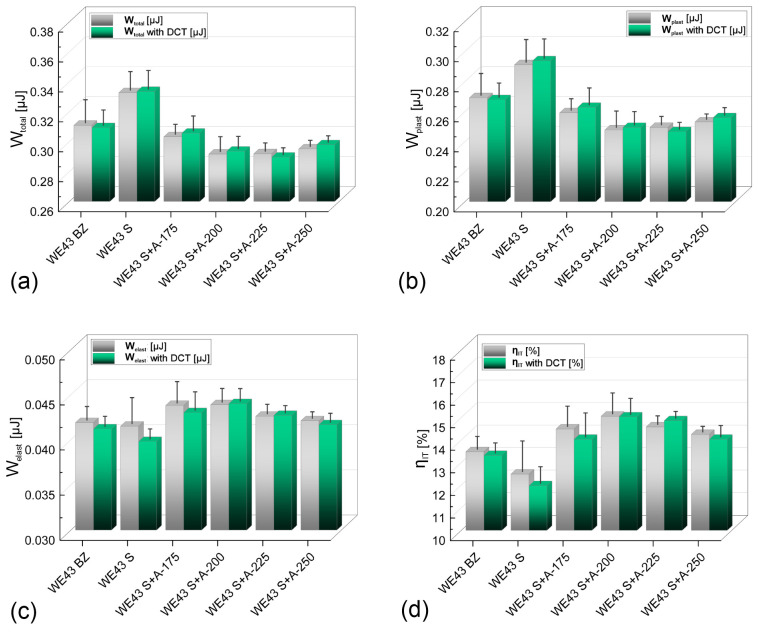
Total work of indentation W_total_—(**a**); the work of plastic deformation W_plast_—(**b**); the work of elastic deformation W_elast_—(**c**) and percentage of elastic deformation work η_IT_—(**d**) of as-received WE43 alloy; after deep cryogenic treatment; and after solution treatment with aging in temperatures of 175–250 °C (with and without cryogenic treatment).

Figure 7 presents the dependence of hardness (H_IT_*)* and Young’s modulus (E_IT_) on the applied heat treatment, including aging temperature, and examples of indentation curves. Figure 8 shows changes in the work of indentation (total W_total_, plastic deformation W_plast_, and elastic deformation W_elast_) of the WE43 alloy and the percentage of elastic deformation work η_IT_.

Analysis of the microindentation test results showed that the introduction of a deep cryogenic treatment and the selection of an appropriate aging temperature for the WE43 alloy led mainly to an increase in the hardness of the alloy (Figure 7a). The increasing trend is observed for temperatures between 175 °C and 225 °C. The results show that for the magnesium alloy aged at 250 °C, there is a decrease in hardness. It can also be observed that the deep cryogenic treatment introduced to the process at each aging temperature resulted in an increase in hardness as well. The most advantageous results were recorded for an alloy subjected to solution treatment at 545 °C/8 h and aged at 225 °C for 24 h with DCT (WE43 S + A225 with DCT). The hardness for this variant (1.157 GPa) increased by approx. 30% compared to the alloy in the initial state (WE43 BZ, 0.908 GPa). Increasing the temperature to 250 °C provides only an approx. 15% increase in hardness compared to the as-received alloy. The introduction of sub-zero treatment, in turn, increases the hardness by approx. 8% compared to the alloy subjected to heat treatment without DCT. Similar dependencies can be observed for Young’s modulus (Figure 7b). Increasing the aging temperature within the range of 175–225 °C results in a gradual increase in the Young’s modulus, with a maximum of 10% increase compared to the as-received material.

Recording the instrumental indentation curves (Figure 7c) made it possible to calculate the work of total deformation W_total_ and its components: the work of plastic deformation W_plast_ and the work of elastic deformation W_elast_ (Figure 8). The analysis shows that an adequately selected alloy aging temperature increases the deformation resistance of the WE43 alloy studied. The increase is observed through changes in parameters such as the depth of indenter penetration, surface area, and volume of the indent, which leads directly to a decrease in the value of the total indentation work. For an alloy subjected to solution treatment and aged at 225 °C for 24 h with DCT (WE43 S + A225 with DCT), the total work of indentation decreased by approx. 7–10% compared to the material in its initial state (WE43 BZ). Taking into account the increasing hardness and Young’s modulus, this indicates the most favorable resistance to deformation. Solution treatment of the alloy, in turn, leads to an increase in the total work of indentation and its plastic component W_plast_, which is directly related to the observed decrease in hardness of the tested alloy. Aging and aging combined with sub-zero treatment lead to small increases in the elastic parameters of the material, such as the work of elastic deformation, W_elast_, and Young’s modulus.

Similar results were obtained for, e.g., the magnesium alloy AZ31 [29,40] or the Mg-Zn-Gd alloy [34,41]. Among other things, an increase in the mechanical and tribological properties was found where precipitation hardening or precipitation hardening combined with sub-zero treatment was applied. It was found that the yield stress was higher after T6 treatment, and the elongation was higher after T6 treatment combined with deep cryogenic treatment. Similarly to this paper, the effect of DCT on the microstructure of the alloy, including an increase in the number of precipitates, was also observed.

The improvement in the properties of the WE43 alloy, observed with appropriately selected heat treatment parameters, such as temperature and time of solution treatment and aging combined with deep cryogenic treatment (DCT), constitutes an effective method for improving the properties of the WE43 magnesium-rare earth alloy.

## 4. Conclusions

In this study, the aging temperature of the WE43 alloy was selected for precipitation hardening and precipitation hardening combined with deep cryogenic treatment. Twelve different alloy variants were obtained using the initial samples, and the results of their microscopic examination (LM, SEM) with a quantitative analysis, X-ray diffraction tests (XRD), microanalysis of the chemical composition (EDS) with distribution maps of elements, and instrumental indentation tests (IIT) are presented. The research conducted by the authors leads to the following conclusions:

1. There were no notable disparities in the microstructure of the WE43 magnesium alloy between the initial state and after undergoing deep cryogenic treatment. In both cases, the magnesium alloy had a fine-grained structure.
2. Solution treatment of the WE43 alloy at 545 °C for 24 h resulted in a 100–120-fold increase in the grain size and most of the precipitates dissolved in the matrix, especially after applying deep cryogenic treatment after solution treatment, where a more than 10-fold reduction in the number of precipitates was observed compared to the material in its initial state. On the diffractograms of the alloy, phases such as α-Mg and La_0.5_RE_0.5_ were identified. Microanalysis of the chemical composition also indicates a homogeneous composition of the alloy after solution treatment.
3. The aging of the alloy carried out in the temperature range of 175–225 °C for 24 h, especially using cryogenic treatment in liquid nitrogen (−196 °C/24 h), led to a reduction in the grain size compared to the samples not subjected to deep cryogenic treatment. For the WE43 alloy aged at 250 °C/24 h, it can be observed that the grain size increases significantly compared to the alloy aged at 225 °C/24 h in the case of both heat treatments (without and with DCT).
4. During aging, the amount of β-phase strengthening precipitates increases. For temperatures of 175–225 °C, it was observed that as the aging temperature increased, there was a maximum 5-fold increase in the amount of precipitates compared to the alloy after solution and sub-zero treatment. These precipitates are of fine size, and only such can result in the strengthening of the material. The most advantageous results were obtained for the alloy aged at 225 °C/24 h and subjected to deep cryogenic treatment (S + A-225 with DCT). Due to lower aging temperatures, it is possible to prevent grain re-growth, which adversely affects the properties of magnesium alloys with rare earth metals.
5. After aging and after aging with sub-zero treatment, the following phases were identified: Mg, La_0.5_RE_0.5_, Mg_3_RE, Mg_2_Nd, MgRE, MgY, and Y_0.65_RE_0.35_, and microanalysis of the phase composition confirmed the presence of phases with yttrium as well as zirconium-rich areas.
6. The positive changes in the WE43 alloy structure achieved by lowering the aging temperature lead to improvements in the micromechanical properties of the test material. The most advantageous results were recorded for the alloy subjected to solution treatment, aged at 225 °C for 24 h, and subjected to deep cryogenic treatment (WE43 S + A225 with DCT). Its hardness increased by approx. 30%, a 10% increase in Young’s modulus was recorded, and the elastic properties of the alloy were improved compared to the initial state (as received). Furthermore, an adequately selected temperature of aging increases the resistance to deformation of the WE43 alloy.
7. Analysis of the results of tests on the alloy aged at 250 °C reveals unfavorable changes in its microstructure and a decrease in its micromechanical properties, indicative of a phenomenon known as alloy overaging.
8. Deep cryogenic treatment combined with a properly conducted precipitation hardening process is an effective method for improving the properties of rare earth metal alloys.

## Figures and Tables

**Table 1:** Certified chemical composition of the WE43 alloy in the as-delivered condition.

Content of Components, wt.-%						
Y	Zr	Zn	Mn	Cu	RE	Mg
3.8	0.5	0.01	0.02	0.002	5.3	Balance

**Table 2:** Complex heat treatment of the WE43 magnesium alloy.

Sample	Heat Treatment Stages		
	Solution Treatment (S)	Deep Cryogenic Treatment (DCT)	Aging (A)
WE43—BZ (initial state)	-	-	-
WE43—BZ with DCT	-	−196 °C/24 h	-
WE43—S	545 °C/8 h	-	-
WE43—S with DCT	545 °C/8 h	−196 °C/24 h	-
WE43—S + A-175	545 °C/8 h	-	175 °C/24 h
WE43—S + A-175 with DCT	545 °C/8 h	−196 °C/24 h	175 °C/24 h
WE43—S + A-200	545 °C/8 h	-	200 °C/24 h
WE43—S + A-200 with DCT	545 °C/8 h	−196 °C/24 h	200 °C/24 h
WE43—S + A-225	545 °C/8 h	-	225 °C/24 h
WE43—S + A-225 with DCT	545 °C/8 h	−196 °C/24 h	225 °C/24 h
WE43—S + A-250	545 °C/8 h	-	250 °C/24 h
WE43—S + A-250 with DCT	545 °C/8 h	−196 °C/24 h	250 °C/24 h

BZ—initial state; DCT—deep cryogenic treatment; S—solution treatment; A—Aging.

**Table 3:** Grain size G, average grain area S_A,_ and volume fraction of intermetallic phases V_V_ of the WE43 alloy in the initial state after different heat treatment variants (as per the ASTM E112 standard).

Sample Designation	ASTM Grain Size Number G	Mean Grain Area S_A_ [µm²]	Volume Fraction of Intermetallic Phases V_V_ [%]
WE43—BZ (initial state)	11.12	58	9.03
WE43—BZ with DCT	10.78	74	7.34
WE43—S	4.19	7048	1.84
WE43—S with DCT	4.52	5638	0.84
WE43—S + A-175	4.10	7523	2.49
WE43—S + A-175 with DCT	4.90	4311	2.69
WE43—S + A-200	3.89	8691	3.66
WE43—S + A-200 with DCT	4.17	7184	2.54
WE43—S + A-225	4.28	6665	4.67
WE43—S + A-225 with DCT	4.35	6334	4.38
WE43—S + A-250	4.21	6952	7.73
WE43—S + A-250 with DCT	4.13	7351	12.35

## Data Availability

The data presented in this study are available on request from the corresponding author.
